# Eosinophilic granulomatosis with polyangiitis exhibits T cell activation and IgG4 immune response in the tissue; comparison with IgG4-related disease

**DOI:** 10.1136/rmdopen-2021-002086

**Published:** 2022-03-08

**Authors:** Satoshi Kubo, Ryuichiro Kanda, Aya Nawata, Yusuke Miyazaki, Akio Kawabe, Kentaro Hanami, Keisuke Nakatsuka, Kazuyoshi Saito, Shingo Nakayamada, Yoshiya Tanaka

**Affiliations:** 1 First Department of Internal Medicine, University of Occupational and Environmental Health, Japan, Kitakyushu, Fukuoka, Japan; 2 Department of Pathology and Oncology, University of Occupational and Environmental Health, Japan, Kitakyushu, Fukuoka, Japan; 3 Department of Rheumatology and Diabetology, Wakamatsu Hospital of the University of Occupational and Environmental Health, Kitakyushu, Fukuoka, Japan; 4 Department of Internal Medicine, Fukuoka Yutaka Central Hospital, Nogata, Fukuoka, Japan; 5 Department of Clinical Immunology and Rheumatology, Tobata General Hospital, Kitakyushu, Fukuoka, Japan

**Keywords:** autoimmune diseases, granulomatosis with polyangiitis, inflammation

## Abstract

**Objective:**

To study the pathophysiological differences of EGPA and IgG_4_-related disease (RD) by clarifying their clinical, pathological and immunological features.

**Methods:**

Clinical and pathological findings were compared in patients with EGPA and IgG_4_-RD. Peripheral blood mononuclear cells were used for comprehensive flow cytometric analysis.

**Results:**

An elevation of the IgG4 level was found in all EGPA cases, with the accompanying pathological findings of lymphocytic infiltration and fibrosis observed in 30.8% patients, and the elevation of IgG_4_/IgG ratio in 61.5% patients. However, actual IgG_4_ levels, as well as the degree of the infiltration of IgG_4_-positive plasma cells, were still higher in patients with IgG_4_-RD than patients with EGPA. Examination by ACR/EULAR classification criteria showed only 13.6% of the EGPA patients met entry criteria, while all of them met the exclusion criteria. In regard to the immunophenotyping, EGPA patients had increases in activated CD4 and CD8 T cells compared with the healthy controls. However, no such similar changes occurred in IgG_4_-RD patients. On the other hand, both the EGPA and IgG_4_-RD patient groups had correlated increased plasmablasts and Tfh. These results indicate the presence of two axes: namely, the activation of T cells and that of B cells. Both axes are present in EGPA, but the T cell activation axis was not observed in IgG_4_-RD.

**Conclusions:**

The elevation of serum IgG_4_ as well as pathological IgG_4_ infiltration are not specific. Meanwhile, EGPA and IgG4-RD differ in immunological phenotypes, indicating the possible importance of the predominant activation of T cells in the development of vasculitis.

**Key messages**

**What is already known about this subject?**

- EGPA and microscopic polyangiitis (MPAs) show a common vasculitis that affects the small blood vessels, and EGPA and IgG4-related disease (RD) have common features including a history of allergic disease, elevated serum IgE, and eosinophilia. In addition, the increase of serum IgG4 in EGPA patients and its subsequent decrease on treatment have already been made known.

**What does this study add?**

- Elevation of IgG4 in both serum and tissues are not highly specific to patients with IgG4-RD. Both activated CD4 T cells and activated CD8 T cells (ie, T cell activation axis) and plasmablasts and follicular helper T cells (ie, B cell activation axis) are elevated in EGPA patients, while only the B cell activation axis is seen in patients with IgG4-RD. Specifically, the T cell activation axis is not seen in patients with IgG4-RD.

**How might this impact on clinical practice or further developments?**

- T cell activation is important for the development of the pathology of vasculitis, and the lack of a T cell activation axis would explain the clinical differences between EGPA and IgG4-RD. Additionally, these results increase the basic scientific knowledge concerning the clinical efficacy of B cell targeted therapy against IgG4-RD.

## Introduction

Eosinophilic granulomatosis with polyangiitis (EGPA) is one of the antineutrophil cytoplasmic antibody (ANCA)-associated vasculitis (AAV) diseases that presents, along with eosinophilia, with an allergic predisposition, with eosinophil infiltration in tissues, and with clinical presentations of vasculitis such as purpura and peripheral neuropathy.^1^ IgG4-related disease (IgG4-RD) is characterised by elevated IgG4, but its pathogenesis remains unknown.^2^ The comprehensive diagnostic criteria for IgG-RD, which have been widely used in many countries and have been published by the Japanese IgG4-RD team organised by the Ministry of Health, Labor and Welfare of Japan, was further published and revised.^3 4^ Although these criteria are not necessarily highly specific, characteristics of this disease are captured in a well-balanced manner from three points of view: clinical signs, serological findings, and pathological findings. Furthermore, the American College Rheumatology (ACR)/(EULAR) classification criteria for IgG4-RD, which have been examined by several centres around the world, were established in 2019 mainly by Stone *et al*.^5 6^ These classification criteria are of very high specificity of 97%–99%, and therefore, contribute greatly both to clinical and epidemiological studies of IgG4-RD, as well as to basic science. Diagnostic criteria and classification criteria have different roles. However, both are beneficial and are likely to be continuously used.

There are several clinical similarities between EGPA and IgG4-RD. For example, a history of allergies appears in 30% and 50% of the patients with EGPA and IgG4-RD respectively.^7 8^ In addition, eosinophilia and elevated IgE are commonly seen in both diseases.^9 10^ More strikingly, the increase of IgG4 occurs in EGPA and its decrease with treatment has subsequently also been made known.^11^ Elevated serum IgG4 and the infiltration of IgG4 positive plasma cells in the tissue are core concepts of IgG4-RD. Therefore, they are sometimes reported as overlapping diseases because of their common characteristic of elevated IgG4.^12–18^ We have also reported a case of EGPA as a mimicker of IgG4-RD.^19^ These reports commonly suggest an existing overlapping pathogenesis in the disease course of EGPA and IgG4-RD. However, EGPA and IgG4-RD are completely different diseases—one is an AAV and the other is a mass-forming disease. This confusion is due to the absence of clarified differences in the pathogenesis between the two; therefore, its elucidation is of great clinical and pathological significance. In this study, we used clinical measurements, pathology assessments, and immunophenotyping with flow cytometry in untreated, newly diagnosed patients to clarify the similarities and differences between the two diseases, thereby exploring and elucidating their pathologies.

## Patients and methods

### Patients

This was conducted as a multicentre study. We enrolled in this study, patients who were both untreated and newly diagnosed with EGPA, IgG4-RD, between March 2013 and March 2018 from four facilities (including University of Occupational and Environmental Health Japan, Wakamatsu Hospital, Tobata General hospital and Kitakyushu General Hospital). In addition, newly diagnosed microscopic polyangiitis (MPA) patients were also enrolled as a control vasculitis group for the immunophenotyping portion of this study. Patient diagnoses were made by at least three doctors who are specialists in the field of Rheumatology. As a result, all subjects fulfilled either the classification criteria or diagnostic criteria.^4 20 21^ The Human Ethics Review Committee of our university reviewed and approved this study, including the collection of peripheral blood samples. Each subject provided a signed consent form.

### Diagnostic criteria and classification criteria for IgG4-RD

Comprehensive diagnostic criteria for IgG4-RD^3 4^ and the ACR/EULAR classification criteria^5 6^ were used for the comparison of EGPA and IgG4-RD. Shortened, straightforward descriptions of comprehensive diagnostic criteria for IgG4-RD are located in the online supplemental table S1. ACR/EULAR classification criteria consist of entry criteria, exclusion criteria and inclusion criteria using a scoring system. Briefly, IgG4-RD is classified if the case meets the entry criteria, no exclusion criteria are present, and the total points are ≥20.

10.1136/rmdopen-2021-002086.supp2 Supplementary data

### Clinical measurement

The laboratory tests included serum IgG, IgG4 and ANCA in addition to a general comprehensive laboratory test. Whole-body CT scan was perfomed in each of the patients to investigate specific organ involvement.

### Pathological assessment

All biopsy or resected samples were embedded in paraffin and stained with H&E and Masson-Trichrome (MT) in both diseases. Lymphoplasmacytic infiltration and eosinophilic infiltration were evaluated by H&E staining. Typical fibrosis of IgG4RD including storiform fibrosis and bird’s eye pattern fibrosis (kidney) was assessed by MT staining. Antibodies against IgG and IgG4 were used for immunohistochemical staining. IgG_4_-positive plasma cell infiltration was counted, and ration of IgG4/IgG were calculated. The pathological assessment was done by two certified pathologists.

### Immunophenotyping analysis

Peripheral blood immunophenotyping was performed by comprehensive eight-colour flow cytometric analysis, proposed by the National Institutes of Health/Federation of Clinical Immunology Societies as a Human Immunology Project, with some necessary modifications for detecting Tfh cells.^22^ Briefly, the phenotyping of immune cell subsets was conducted as described previously.^23 24^ The peripheral blood mononuclear cells were incubated in blocking buffer and then suspended in FACS solution with fluorochrome-conjugated monoclonal antibodies. Data collection was performed with a FACSVerse (Becton-Dickinson, San Jose, CA, USA) and further analysed with Flow Jo software (Tree Star, Ashland, Oregon, USA). As part of our immunophenotyping, we further included MPA patients as a control for vasculitis.

### Statistical analysis

Continuous data were expressed as the mean±SD, and categorical data expressed as the number (%). Baseline clinical characteristics and the proportion of immune cell subsets between groups were compared using the Mann-Whitney U test. The optimal cut-off value to distinguish two diseases was calculated using receiver operator characteristic (ROC) curve analysis. For easy exploration and visualisation of immunophenotyping data, we used principal component analysis (PCA) to statistically aggregate items, reducing the number of observed variables into a smaller number of principal components (PC) and reducing the dimensionality of the immunophenotyping data. PCA was performed as described before.^23^ Briefly, the values for PC were calculated in individual patients. We selected two eigenvectors with the highest eigenvalues as PC1 (eigenvalue 4.3) and PC2 (eigenvalue 3.5) based on each contribution rate. The statistical correlations among immune cell subset proportions were calculated by the Spearman’s rank correlation coefficient. To draw edges between each cell subsets with positive correlations, Cytoscape V.3.9.0 was used. The level of significance was set at p<0.02. Each circle size was defined from the proportion of each cell subset in comparison to the healthy controls. All analyses were conducted using IBM SPSS Statistics V.22.0 (IBM) or JMP V.16.0 (SAS Institute).

## Results

### Clinical characteristics

Twenty-two EGPA patients and 20 IgG4-RD patients were enrolled in this study. The mean age was comparable, but the proportion of woman was dominant in the EGPA group (table 1). Glandular manifestation was often seen in patients with IgG4-RD, while there were no EGPA patients with glandular manifestation. On the other hand, EGPA patients showed symptoms such as skin rashes and peripheral neuropathy due to vasculitis, but patients with IgG4-RD exhibited few to any similar findings (table 1). Concomitant ear-nose-throat involvement was comparable between EGPA and IgG4-RD.

**Table 1:** Baseline characteristics and disease activity

	Eosinophilic granulomatosis with polyangitis	IgG_4_-related disease	P value
Age, years	60.7±2.8	61.5±3.6	0.87
Female, n (%)	18 (81.8)	8 (40.0)	0.01
Glandular manifestation, n (%)	0 (0.0)	15 (75.0)	<0.001
Ear-nose-throat involvement, n (%)	14 (63.6)	9 (45.0)	0.23
Skin involvement, n (%)	15 (68.2)	0 (0.0)	<0.001
Lung/lower airway tract involvement			
Interstitial lung disease, n (%)	2 (9.1)	4 (20.0)	0.31
Migratory pulmonary infiltrates, n (%)	2 (9.1)	0 (0.0)	0.17
Pleural effusion, n (%)	5 (22.7)	0 (0.0)	0.02
Neurological manifestations			
CNS involvement, n (%)	0 (0.0)	2 (10.0)	0.13
Peripheral neuropathy, n (%)	19 (86.4)	1 (5.0)	<0.001
Gastrointestinal involvement, n (%)	4 (18.2)	0 (0.0)	0.045
Pancreas involvement, n (%)	0 (0.0)	2 (10.0)	0.13
Heart involvement, n (%)	2 (9.1)	0 (0.0)	0.17
Renal involvement, n (%)	4 (18.2)	4 (20.0)	0.89
Blood count			
White cell count, / x10^ˆ9^/L	32.0±9.5 x10^ˆ9^/L	6.1±0.5 x10^ˆ9^/L	0.01
Eosinophil granulocyte, /μL	18296.4±5804.7	487.2±133.7	0.01
Immunoglobulin			
IgG, mg/dL	2367.6±190.7	2992.4±348.7	0.13
IgA, mg/dL	263.0±23.5	231.6±33.7	0.44
IgM, mg/dL	149.7±14.3	91.9±15.7	0.01
IgE, mg/dL	4466.8±1938.2	1563.5±964.5	0.25
IgG_4_, mg/dL	516.2±47.9	1063.0±217.7	0.02
IgG_4_/IgG ratio (%)	22.2	30.9	0.03
Blood biochemistry			
C3, mg/dL	119.2±5.8	81.7±7.8	<0.001
C4, mg/dL	24.6±2.3	16.3±2.3	0.01
CH50, U/mL	51.4±3.3	39.3±5.0	0.05
CRP, mg/dL	6.7±1.2	0.6±0.4	<0.001
RF, U/mL	207.1±44.3	51.5±19.0	<0.001
sIL-2R, U/mL	2688.0±338.2	1245.6±295.0	<0.001
Positive for MPO-ANCA, n (%)	9 (40.9)	0 (0.0)	<0.001
Positive for PR3-ANCA, n (%)	1 (4.5)	0 (0.0)	0.4

Glandular manifestation includes involvement of bilateral lacrimal gland, submandibular gland and parotid gland. Results are shown as mean±SEM unless stated otherwise.

CNS, central nervous system; CRP, C reactive protein; MPO-ANCA, myeroperoxidase anti-neutrophil cytoplasmic antibody; PR3-ANCA, proteinase3 anti-neutrophil cytoplasmic antibody; RF, Rheumatoid factor.

### Haematological findings

The results of the laboratory tests are shown in table 1. There were a substantial number of differences between EGPA patients and IgG4-RD patients as expected. In particular, the mean eosinophil counts were 18 296.4 /µL in EGPA and 487.2 /µL in IgG4-RD (table 1). Meanwhile, IgG4-RD patients showed an increase in the level of serum IgG4 (1063 mg/dL), higher than that of the EGPA patients (516.2 mg/dL). In addition, hypocomplementaemia was observed only in patients with IgG4-RD, but no increased inflammatory response was observed. On the other hand, IgG, IgA and IgE were comparable between EGPA and IgG4-RD. Moreover, if we focused on the abnormal values in regards to eosinophil count, we found that eosinophilia was present in both EGPA patients and IgG4-RD patients (table 1). Namely, although the laboratory findings exhibited varying degrees of abnormality, several of the findings themselves did overlap in both diseases.

### Histological findings

**Figure 1:**
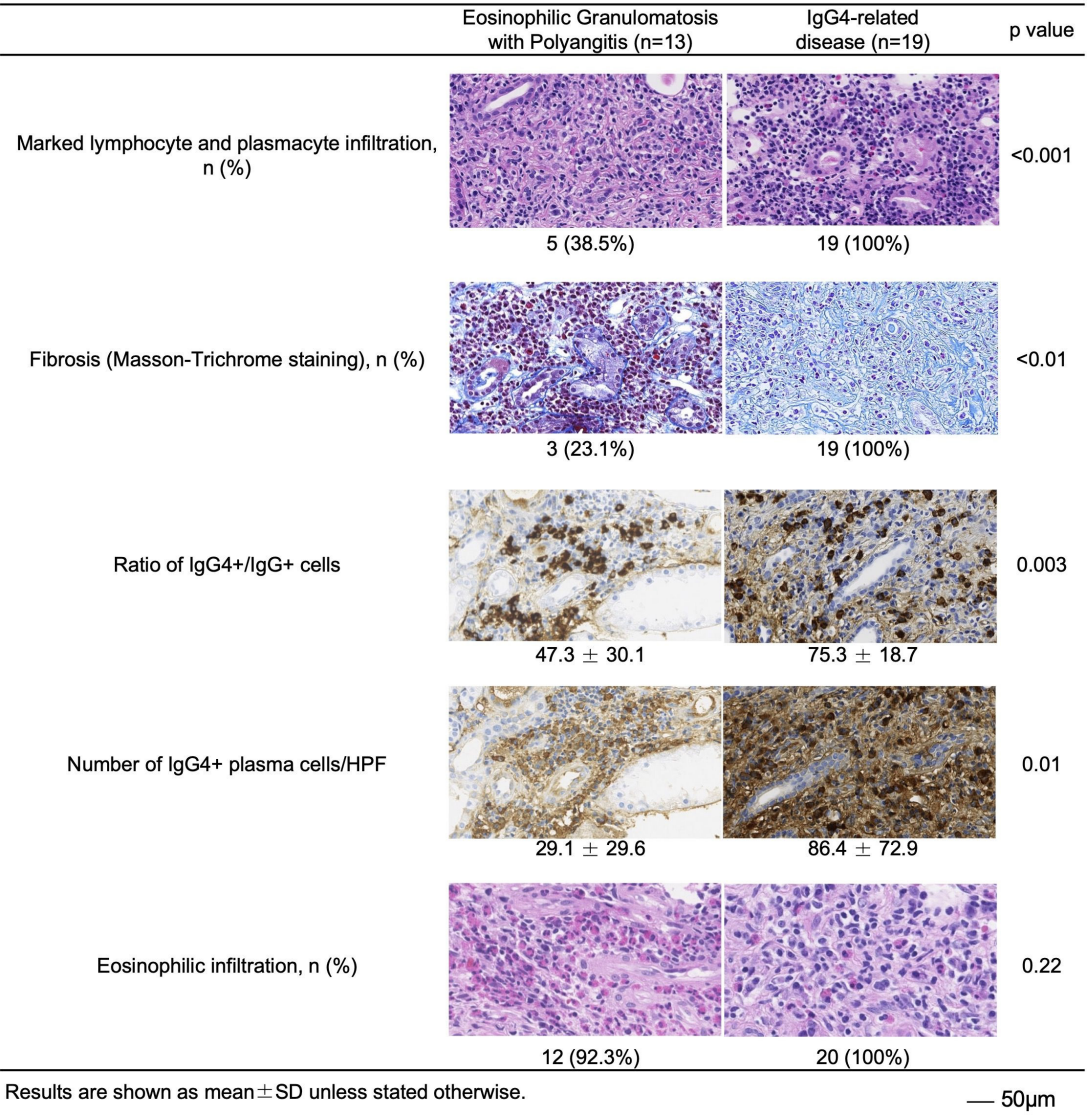
Histological findings between EGPA and IgG4-RD. The sites of organ involvement were evaluated by each item. Lymphocyte, plasmacyte and eosinophilic infiltration were evaluated by H&E staining. IgG and IgG_4_ were stained by immunohistochemistry. Fibrosis was evaluated by Masson-Trichrome staining. RD, related disease; EGPA, Eosinophilic granulomatosis with polyangiitis; HPF, high-power field.

In order to investigate the differences in the pathogenesis between EGPA and IgG4-RD, we assessed the pathological findings. The number of IgG4 positive plasma cells and the ratio of IgG4^+^/IgG^+^ cells were higher in patients with IgG4-RD (figure 1). However, although the degree was different, the number of IgG4 positive plasma cells and ratio of IgG4^+^/IgG^+^ cells at the site of organ involvement were also increased in patients with EGPA. Marked infiltration of lymphocytes and plasma cells and fibrosis are one of the pathological features of IgG4-RD, and these findings are observed in all cases of IgG4-RD. However, these findings were also found in about 20% to 40% of patients with EGPA. Additionally, there was no difference in the eosinophilic infiltration between the two diseases (figure 1). In other words, it was clarified that there is pathological homology between IgG4-RD and EGPA. Despite these findings, the severity between the two diseases was different, and the infiltration of IgG4-positive plasma cells was more pronounced in patients with IgG4-RD.

### Comprehensive clinical diagnostic criteria for IgG4-RD

**Figure 2:**
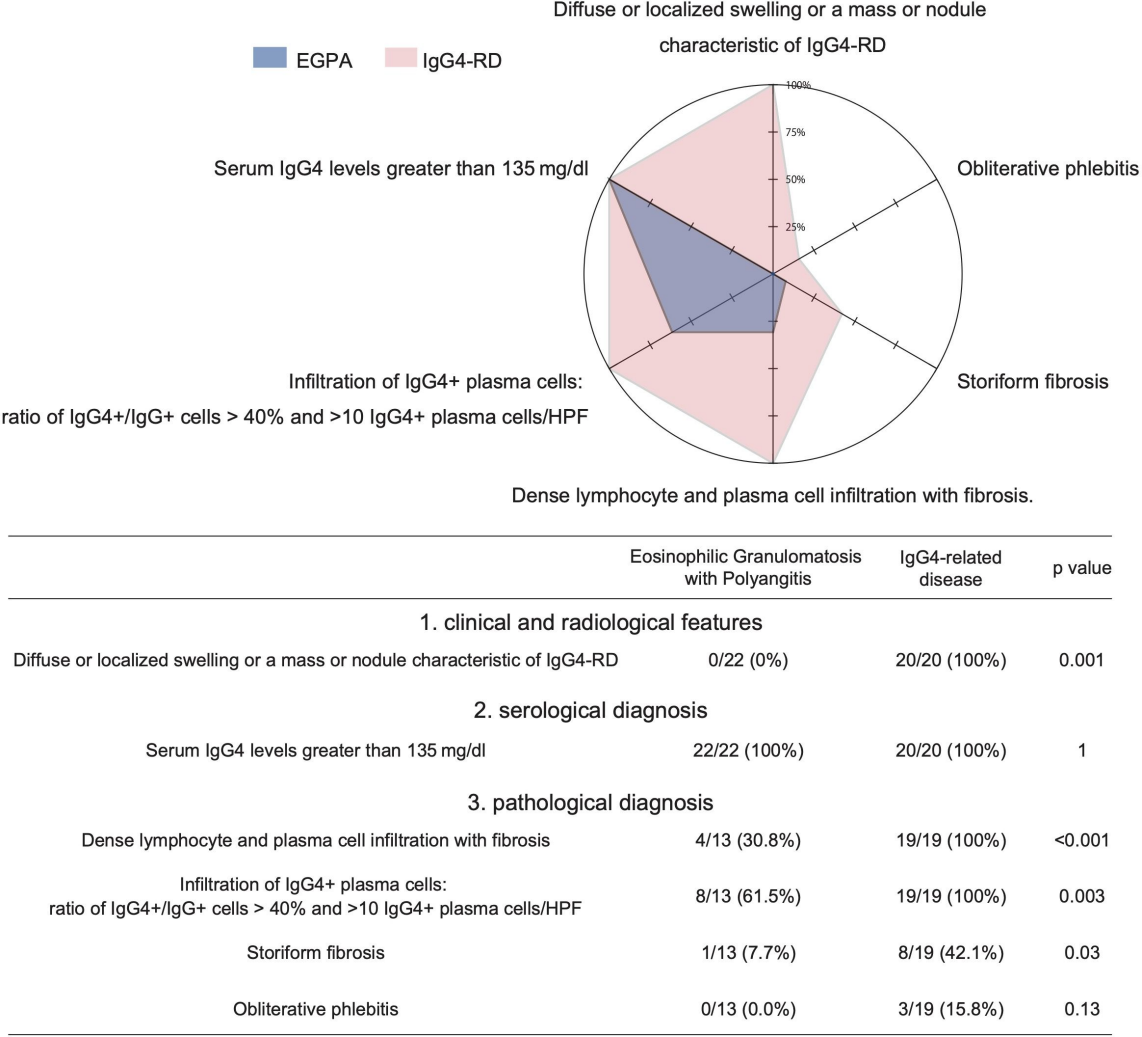
Fulfilling comprehensive clinical diagnostic criteria for IgG4-RD. The proportion of cases fulfilling each item in the criteria. RD, related disease; EGPA, Eosinophilic granulomatosis with polyangiitis; HPF, High-power field.

Comprehensive diagnostic criteria for IgG4-RD are widely used for the diagnosis of IgG4-RD in clinical practice.^3 4^ These diagnostic criteria well capture the clinical characteristics of IgG4-RD. Therefore, we investigated the clinical overlaps between EGPA and IgG4-RD found and described in this report, based on those criteria (figure 2). The criterion of increased serum IgG4 was satisfied in all cases, even in patients with EGPA. The pathological findings also overlapped between EGPA and IgG4-RD. Namely, dense lymphocyte and plasma cell infiltration with fibrosis was seen in 30.8% and an increased ratio of IgG4^+^/IgG^+^ plasma cells in 61.5% of EGPA patients (figure 2). On the other hand, specific pathological findings such as storiform fibrosis and obliterative phlebitis were seen in 42.1% and 15.8% of IgG4-RD patients respectively, while these same findings were rarely seen in patients with EGPA. Notably, the clinical and radiological features of mass-forming lesions, which were seen in all IgG4-RD patients, were not detected in EGPA patients.

### The 2019 ACR/EULAR Classification Criteria for IgG4-related disease

**Figure 3:**
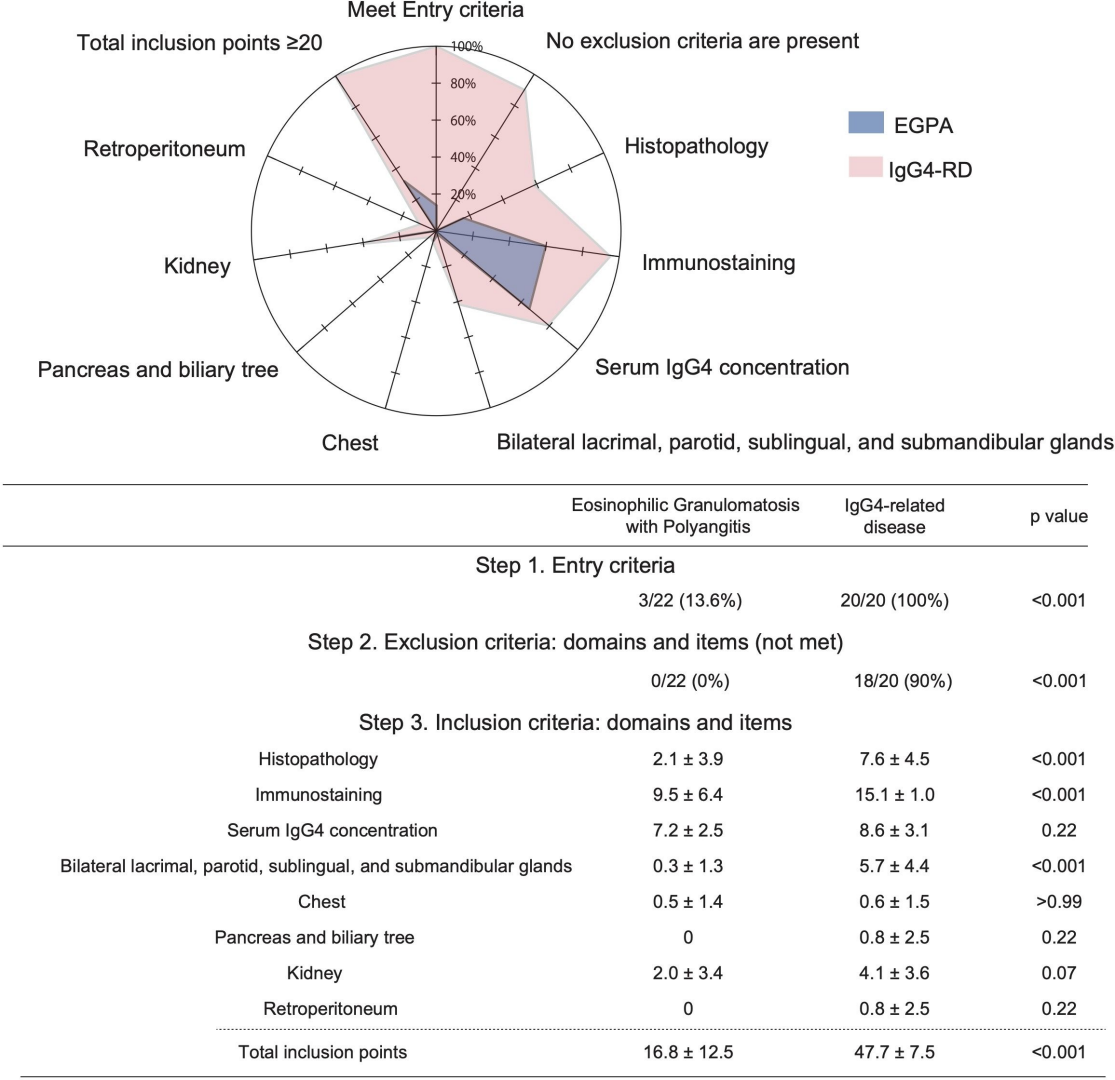
Fulfilling the 2019 ACR/EULAR classification criteria for IgG_4_-related disease (RD). The graph shows the proportion of EGPA and IgG_4_-RD cases respectively fulfilling the entry criteria and not fulfilling the exclusion criteria. for the inclusion criteria, this list states the mean score in EGPA and IgG_4_-RD cases regarding each item. The mean score as a percentage of the full score for each criterion is shown in the graph at the top. The mean for the total inclusion score is listed as the end of the table. ACR/EULAR. EGPA, Eosinophilic granulomatosis with polyangiitis.

As discussed previously, the classification criteria for IgG4-RD were established by ACR/EULAR in 2019.^5 6^ These criteria use a scoring system for each organ and have extremely high specificity. We thus investigated the clinical findings to see how many EGPA cases fulfilled these criteria. Only 13.6% of EGPA cases met the entry criteria, and all cases met the exclusion criteria (figure 3). In other words, all patients with EGPA were eliminated as IgG4-RD before moving forward to the inclusion criteria. If we investigated whether EGPA patients fulfilled the inclusion criteria of IgG4-RD, the score for serum IgG4 concentration was comparable between EGPA patients and IgG4-RD patients (figure 3). In addition, the score from the immunostaining of IgG and IgG4 in EGPA was two-thirds that of IgG4-RD. On the other hand, obliterative phlebitis and storiform fibrosis were rarely seen in EGPA patients, and thus the score of histopathology was very low. Namely, there was a more pronounced difference between EGPA and IgG4-RD in morphological abnormalities than increased IgG4 or infiltration of IgG4. In terms of the organ involvement, the score for bilateral lacrimal, parotid, sublingual, and submandibular glands showed the biggest differences overall. The involvement of the pancreas and biliary tree or retroperitoneum were specific for IgG4-RD and were not seen in patients with EGPA. IgG4-RD was classified with a score of 20 points or more in the classification criteria, and all the IgG4-RD cases had over 20 points (figure 3). As described above, all EGPA cases were excluded by the entry criteria and exclusion criteria. However, the score was over 20 points in around 30% of EGPA patients if we solely considered the inclusion criteria. The main reason was due to the high scores from immunostaining and serum IgG4.

### Key items for the differential diagnosis between EGPA and IgG4-RD

**Figure 4:**
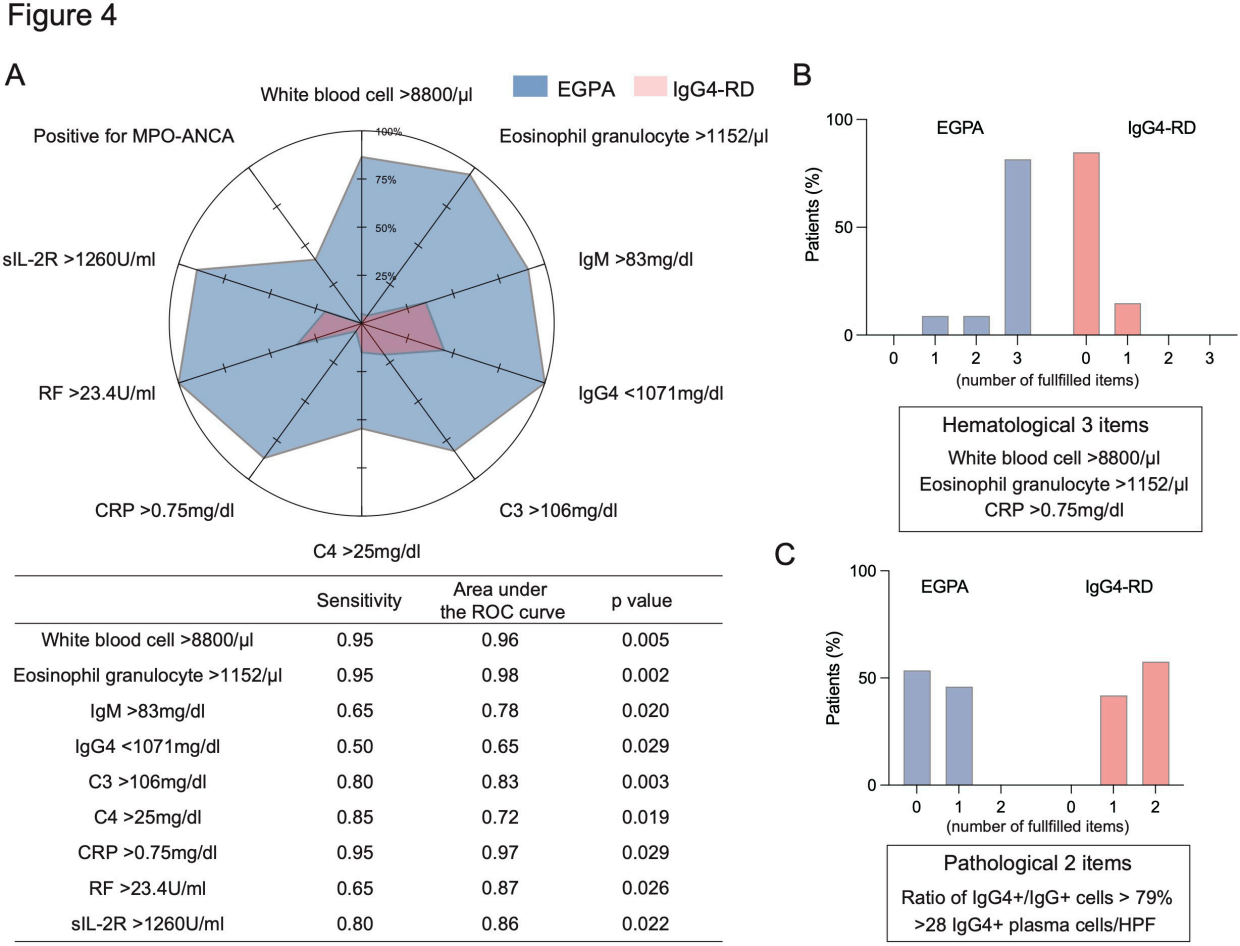
Cut-off value of key items for the differential diagnosis between EGPA and IgG_4_-RD. (A) The proportion of cases fulfilling the cut-off value. The table shows the sensitivity, area under the ROC curve, and p value of each cut-off value. (B, C) the proportion of cases according to the number of fulfilled haematological items (B) pathological items (C) in patients with EGPA and IgG_4_-RD. ANCA, antineutrophil cytoplasmic antibody; CRP, C reactive protein; RD, related disease; ROC, receiver operator characteristic; EGPA, Eosinophilic granulomatosis with polyangiitis; HPF, High-power field; RF, Rheumatoid factor.

Although the affected organ is a key component for the differential diagnosis between EGPA and IgG4-RD, lung and renal involvement were not specific for IgG4-RD (table 1 and figure 3). In the serological findings, eosinophilia and elevated IgG and IgE are one of the characteristics in patients with IgG-4-RD, but these findings were also seen in EGPA. Of note, an increased level of serum IgG4 (>135 mg/dL) was also shown in all EGPA patients (figure 2). Therefore, we next calculated a cut-off value for serological findings to distinguish between these two diseases (figure 4A). Among serological cut-off values, we found that white cell counts (cut-off value: 8.8 x10^ˆ9^/L), eosinophil granulocyte counts (cut-off value: 1152 /µL), and C reactive protein (CRP) (cut-off value: 0.75 mg/dL) showed best area under the ROC curve (AUC). Serological findings above these cut-off values thus are indicative of EGPA and likewise if below, indicate IgG4-RD. On the other hand, the cut-off value for IgG4 (1071 mg/dL) showed relatively narrow AUC (figure 4A). For this value, serological findings below and above the cut-off indicate EGPA and IgG4-RD, respectively. If we picked cut-off values of blood cell counts, eosinophil granulocyte counts, and CRP as markers of EGPA, then there were clear differences between EGPA and IgG4-RD (figure 4B). Namely, none of the IgG4-RD cases fulfilled more than two items. In contrast, a majority of the EGPA cases fulfilled all of the items. For the pathological findings, the differences of morphological abnormalities were more significant than that of IgG4 infiltration (figure 3). In fact, 61.5% of the EGPA patients fulfilled both the ratio of IgG4^+^/IgG^+^ cells > 40% and >10 IgG4 positive plasma cells/High-power field (HPF). If we calculated the cut-off value of the ratio of IgG4^+^/IgG^+^ cells and the number of IgG4 positive plasma cells/HPF for the differential diagnosis between EGPA and IgG4-RD, we found that a higher concentration of IgG4^+^ cells (ratio of IgG4^+^/IgG^+^ cells > 79% and >28 IgG4 positive plasma cells/HPF) were needed (figure 4C). However, these AUCs were not high enough (0.80 and 0.83, respectively), and half of patients with both diseases fulfilled one item (figure 4C). These results suggest that elevation of IgG4 in both serum and tissues is not highly specific.

### Immunophenotyping of EGPA and IgG4-RD

**Figure 5:**
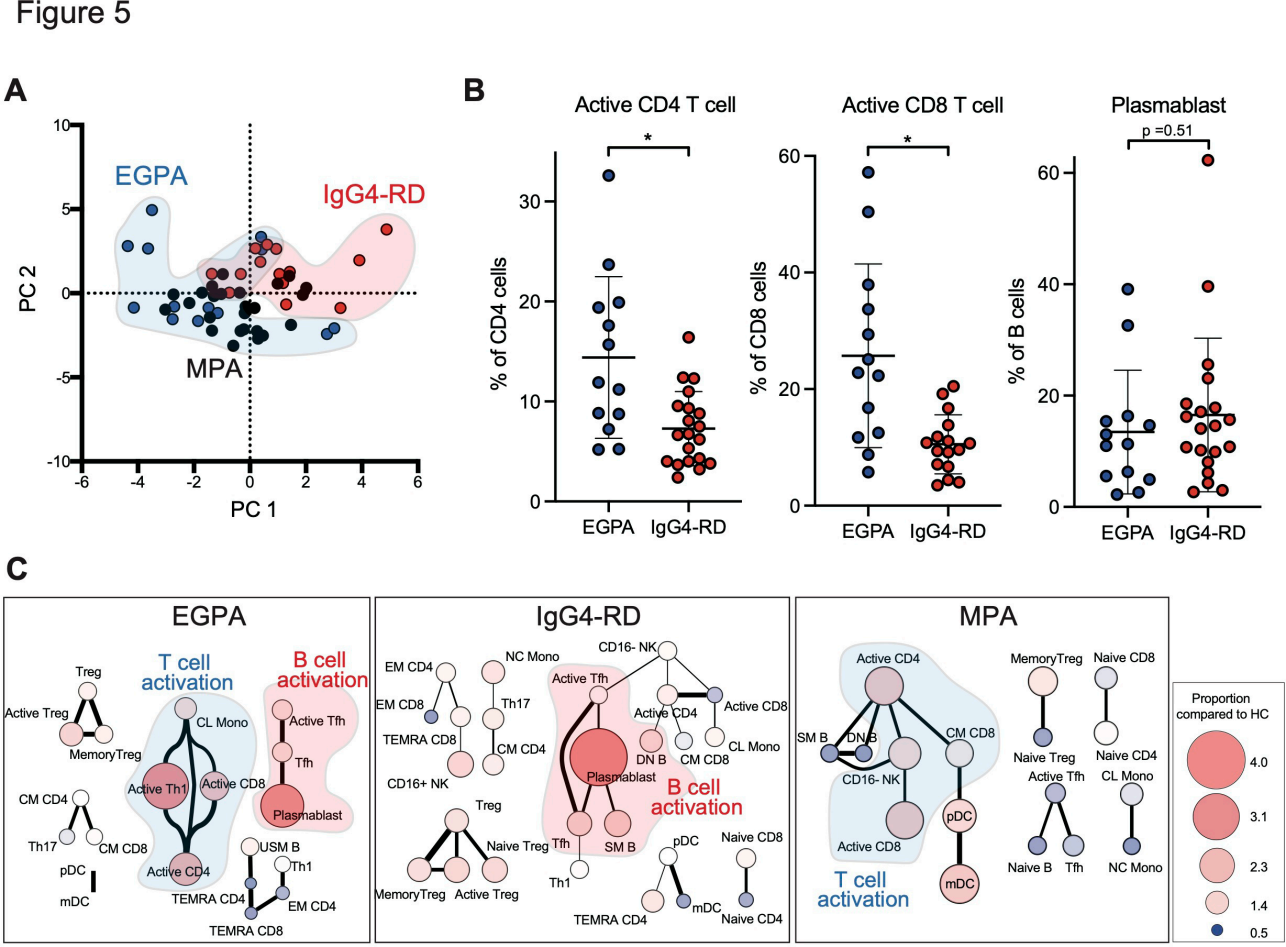
Immunophenotyping of EGPA and IgG4-RD. (A) Te immunophenotype shown by principal component analysis among EGPA, IgG4-RD and MPA. (B) The proportion of activated CD4 T cells, activated CD8 T cells, and plasmablasts between EGPA and IgG4-RD. (C) The visualised model based on the immunophenotyping in EGPA, IgG4-RD and MPA. The colour (blue for decreases and red for increases) and size of circle indicates the ratio of each immune cell proportion in comparison to healthy controls as shown in the right box. Each line shows the statistical positive correlation between immune cells (p value is less than 0.02). Line thickness reflects the value of the Spearman’s rank correlation coefficient (thin for weak and bold for strong). MPA, microscopic polyangiitis; PC, principal components; RD, related disease; EGPA, Eosinophilic granulomatosis with polyangiitis. *p<0.05

There were clinical and pathological overlaps between EGPA and IgG4-RD. We next asked whether there were overlaps and differences in the peripheral blood immunophenotype. As shown by PCA based on the immunophenotyping among them, the phenotype was different between EGPA and IgG4-RD with slightly overlapping characteristics (figure 5A). The immunophenotype of MPA, as a control for vasculitis, was similar to EGPA. When we checked the proportion of each immune cell type among the three diseases (online supplemental figure S1), there were significant differences in the proportion of activated CD4 T cells and activated CD8 T cells between EGPA and IgG4-RD (online supplemental table S2). Namely, populations of both activated CD4 and CD8 T cells were increased in patients with EGPA (figure 5B). On the other hand, the proportion of plasmablasts was comparable (figure 5B). Next, we investigated the correlation among the proportions of these immune cells, and visualised them and found that there were clear differences among these three diseases (figure 5C). Specifically, in EGPA patients, activated T cells and activated CD8 T cells (ie, T cell activation axis) were elevated in a correlated manner, and plasmablasts and follicular helper T cells (ie, B cell activation axis) were also elevated in a correlated manner. These two axes were independent of each other (figure 5C). In patients with IgG4-RD, plasmablasts and follicular helper T cells were also elevated. However, the proportion of activated T cells was relatively lower, and the T cell activation axis was not seen. By contrast, the T cell activation axis was only seen in patients with MPA (figure 5C).

10.1136/rmdopen-2021-002086.supp1 Supplementary data

## Discussion

We conducted this study to examine the similarities and differences in the clinical, pathological, and immunological aspects between EGPA and IgG4-RD, both of which exhibit elevated IgG4 levels, to study the pathologies of these two diseases. In particular, we examined the differences between IgG4-RD and EGPA by using both comprehensive diagnostic criteria for IgG4-RD and ACR/EULAR classification criteria. This allowed us to assess homology and find differences with respect to clinical measurements, pathology assessments and immunophenotyping.

First, distinguishing between EGPA and IgG4-RD by comparing clinical signs is relatively easy, as is evident from the presence of mass-forming lesions as reported herein; that is, the mass-forming lesions were present in all IgG4-RD patients but not in EGPA patients. Mass formation is considered to be a critical clinical feature of IgG4-RD. In addition, only a few glandular symptoms were found in EGPA, with no symptoms found in the pancreas, bile ducts, and retroperitoneal organs. Such differences in target organs may be due to fibrosis and angiopathy, which are fundamental pathologies of EGPA and IgG4-RD in addition to their immunological differences. However, the serum IgG4 level met a criterion of 135 mg/dL, the cut-off value in the comprehensive diagnostic criteria for IgG4-RD, in all EGPA patients, indicating the absence of specificity with regards to elevated IgG4 level.

Pathological findings are among the most important for understanding the pathology and pathogenesis of diseases. There was no difference in eosinophil infiltration between the two diseases. The similar presence of eosinophil infiltration indicates that both are based on allergic predisposition.^25 26^ IL-4 and IL-5 play important roles in IgG4 induction and eosinophilia,^27 28^ indicating that these cytokines are important to both diseases. On the other hand, IgG4 infiltration in the kidney was reported not to be specific to IgG4-RD.^29^ Our data expanded this evidence and showed nonspecific IgG4 positive plasma cell infiltration in EGPA. In IgG4-RD, an improvement of disease state due to treatments is associated with decreased IgG4.^11^ However, pathological significance of IgG4 itself may be low in the pathogenesis of IgG4-RD, considering the lack of specificity of the infiltration of IgG4-positive plasma cells to the lesion area. Investigation of the pathological significance of IgG4 in experimental animal models would be difficult since mice do not have IgG4; therefore, testing using patient samples is incredibly important. The degree of local IgG4 infiltration was higher in IgG4-RD. This also holds true in serum IgG4 levels, suggesting that the production of IgG4 and its infiltration are more pronounced in IgG4-RD. This is likely because the suppression of IgG4 production is balanced in EGPA by the elevation of other cytokines including the Th2 cytokine.

From the view of clinical and pathological findings, we made three chief observations regarding the similarities between EGPA and IgG4-RD: (1) the diagnosis of IgG4-related lung disease and IgG4-related kidney disease need to be more careful made since the lung and kidneys are favoured sites of AAV; (2) an increased level of serum IgG4 (>135 mg/dL) is not specific to IgG4-RD; however, higher IgG4 values (>1100 mg/dL) could increase the specificity towards the diagnosis; and (3) both diseases show eosinophilic infiltration and IgG4 positive plasma cells infiltration at sites of organ involvement. On the other hand, we also could clearly see the differences. The clinical and radiological features of mass-forming lesions are specific findings of IgG4-RD. A substantially increased level of white blood cell counts and serum CRP are not seen in IgG4-RD. In addition, morphological findings such as storiform fibrosis and obliterative phlebitis are specific pathologically.

The results of immunological phenotyping further solidified this finding. Specifically, the Tfh-plasmablast axis is elevated in IgG4-RD to form its pathology, whereas in EGPA the T-cell activation axis, mainly that of Th1, was elevated independently along with the elevation of the Tfh-plasmablast axis. In MPA, a control disease, only T-cell activation was observed. This result, although it must be interpreted with caution, suggests that T-cell activation is important for the development of the pathology of vasculitis. The balance between the factors required for the activation of T cells and those required for the differentiation and activation of B cells could be responsible for the difference in IgG4 levels in IgG4-RD and EGPA, as well as the degree of local IgG4 infiltration.

The limitation of this study is the relatively small number of cases analysed since we only enrolled untreated and newly diagnosed patients. In addition, the measurements of serum cytokines (IL-4, IL-5, IL-10, IL-13 and IL-21), which are important for testing the hypothesis mentioned above, were below the range of the limit of detection in several patients. Th2 and CD4^+^ cytotoxic T lymphocytes^30^ are known to play important roles in the pathogenesis of IgG4-RD. We have not been able to add pathological studies of these cells and cytokines; thus, further studies are desired in the future.

Taken together, this study reaffirmed the importance of the clinical findings of mass formation in IgG4-RD and showed that the elevation of serum IgG4 as well as the pathological IgG4 infiltration are not specific only to IgG4-RD. Meanwhile, EGPA and IgG4-RD differed in immunological phenotypes, indicating a possible importance of the predominant activation of T cells for the development of vasculitis. The balance between the factors required for the activation of T cells and those required for the differentiation and activation of B cells could be responsible for the differences in IgG4-RD and EGPA.

## Data Availability

Data are available on reasonable request. Not applicable.
