# Supplementary material for: Eosinophilic granulomatosis with polyangiitis exhibits T cell activation and IgG4 immune response in the tissue; comparison with IgG4-related disease
Source: RMD Open. 2022 Mar 8;8(1):e002086. doi: 10.1136/rmdopen-2021-002086 (PMC8906049; doi:10.1136/rmdopen-2021-002086)
Supplement: Supplementary data [file rmdopen-2021-002086supp002.pdf]

Table S1. The 2020 Revised comprehensive diagnostic criteria for IgG4-RD

1. clinical and radiological features

   One or more organs show diffuse or localized swelling or a mass or nodule characteristic of IgG4-RD.

   In single organ involvement, lymph node swelling is omitted.

2. serological diagnosis

   Serum IgG4 levels greater than 135 mg/dl.

3. pathological diagnosis

   Positivity for two of the following three criteria:

   (1) Dense lymphocyte and plasma cell infiltration with fibrosis.

   (2) Ratio of IgG4-positive plasma cells /IgG-positive cells greater than 40% and the number of IgG4-positive plasma cells greater than 10 per high powered field

   (3) Typical tissue fibrosis, particularly storiform fibrosis, or obliterative phlebitis

Diagnosis:

Definite: 1) +2) +3)

Probable: 1) +3)

Possible: 1) +2)

Table S2. Differences between EGPA and IgG4-RD

	p value
Naive CD4 T cells	0.02
Activated CD4 T cells	0.02
Activated CD8 T cells	0.01
Myeloid Dendritic cells	<0.001
Plasmacytoid Dendritic cells	<0.001
