# Supplementary material for: Eosinophilic granulomatosis with polyangiitis exhibits T cell activation and IgG4 immune response in the tissue; comparison with IgG4-related disease
Source: RMD Open. 2022 Mar 8;8(1):e002086. doi: 10.1136/rmdopen-2021-002086 (PMC8906049; doi:10.1136/rmdopen-2021-002086)
Supplement: Supplementary data [file rmdopen-2021-002086supp001.pdf]

Supplemental figure S1

A

		Eosinophilic Granulomatosis with Polyangitis (n=13)	IgG4-related disease (n=20)	Microscopic Polyangiitis (n=26)	P value
CD4 T cells	Naive	50.1 ± 15.0	34.7 ± 14.0	46.9 ± 2.9	0.01
	Central memory	35.3 ± 10.5	42.9 ± 11.4	30.7 ± 2.4	0.01
	Effector memory	12.3 ± 9.2	17.3 ± 6.9	17.6 ± 1.7	0.17
	Effector	2.3 ± 1.7	4.9 ± 3.7	5.8 ± 1.2	0.25
	Activated	14.4 ± 8.1	7.3 ± 3.7	11.2 ± 1.6	0.04
CD8 T cells	Naive	50.5 ± 21.2	34.6 ± 19.1	29.1 ± 3.4	<0.01
	Central memory	15.6 ± 8.0	14.7 ± 12.8	18.8 ± 2.3	0.5
	Effector memory	13.4 ± 11.6	16.2 ± 10.9	27.5 ± 2.7	0.01
	Effector	20.6 ± 17.2	34.5 ± 22.3	24.6 ± 3.5	0.09
	Activated	25.7 ± 15.8	10.5 ± 5.1	22.6 ± 2.4	<0.01
CD4 T cell subsets	Th1	21.3 ± 7.4	21.8 ± 7.4	17.7 ± 1.4	0.12
	Activated Th1	5.2 ± 5.1	1.9 ± 1.2	6.7 ± 3.1	0.59
	Th17	10.6 ± 5.0	14.0 ± 5.6	12.9 ± 1.1	0.23
	Activated Th17	2.3 ± 1.8	1.1 ± 0.8	1.4 ± 0.2	0.02
	Treg	4.6 ± 3.0	5.7 ± 1.9	4.6 ± 0.4	0.15
	Activated Treg	2.1 ± 2.1	1.8 ± 0.7	1.8 ± 0.2	0.69
	Naive Treg	1.1 ± 1.5	1.4 ± 0.7	0.6 ± 0.2	0.02
	Memory Treg	3.5 ± 2.4	4.3 ± 1.4	4.0 ± 0.3	0.36
	Tfh	1.3 ± 0.7	1.5 ± 0.6	0.7 ± 0.1	<0.01
	Activated Tfh	0.4 ± 0.3	0.3 ± 0.3	0.2 ± 0.04	<0.01
B cells	Naive	61.9 ± 21.3	59.4 ± 18.4	65.1 ± 3.7	0.6
	IgM memory	12.7 ± 7.8	13.2 ± 4.9	13.2 ± 1.2	0.97
	Class-switched memory	14.2 ± 14.2	20.4 ± 18.0	11.8 ± 2.9	0.16
	Double negative	11.2 ± 9.5	7.1 ± 3.8	10 ± 1.2	0.13
	Plasmablasts	13.5 ± 11.1	16.5 ± 13.8	6.5 ± 2.0	<0.01
Monocytes	Classical	11.4 ± 14.3	8.6 ± 5.3	10.3 ± 1.6	0.64
	Non classical	1.1 ± 1.0	1.1 ± 0.6	1.2 ± 0.2	0.85
Dendritic cells	Myeloid	0.3 ± 0.4	1.0 ± 0.6	1.2 ± 0.4	0.05
	Plasmacytoid	0.03 ± 0.03	0.09 ± 0.04	0.1 ± 0.02	<0.01
NK cells	CD16+ NK cell	10.0 ± 8.5	15.1 ± 13	9.9 ± 2.8	<0.01
	CD16- NK cell	7.5 ± 14.0	1.6 ± 2.0	2.8 ± 1.4	0.14

colors were compared with healthy control

0.1 — 10
